# Zika Virus in a Patient With Cancer: How Much Do We Know?

**DOI:** 10.1200/JGO.2016.003533

**Published:** 2016-04-27

**Authors:** Adriana Hepner, Maria del Pilar Estevez Diz

**Affiliations:** All authors, Hospital Sirio-Libanes, Sao Paulo, Brazil

In the past 20 years, arbovirus infections, caused by RNA viruses transmitted by arthropods, have become a major concern in the Western Hemisphere; examples include dengue, West Nile virus, and chikungunya.^1^ Recently, infection with Zika virus, a flavivirus first discovered incidentally in Uganda in the mid-1940s, has reached pandemic status. Zika virus is transmitted to humans via the bite of an infected *Aedes* species mosquito, but maternal fetal and sexual transmissions have also been described.^2^ The most common symptoms are fever, maculopapular rash, arthralgia (especially in small joints of the extremities), and conjunctivitis, usually beginning 2 to 7 days after the infection. Other commonly reported symptoms are myalgia, headache, and asthenia. Treatment includes rest, fluids to avoid dehydration, and administration of acetaminophen to relieve pain and fever. Patients should be cautioned against the use of aspirin or nonsteroidal anti-inflammatory drugs to reduce the risk of hemorrhage.^2^

In the last 13 months, more than 30 countries have reported active Zika virus transmission in the Americas, Oceania, and Africa.^3^ In April 2015, the first identified case in Brazil was reported. Since then, according to the Brazilian Ministry of Health, between 490,000 and 1,400,000 new cases may have occurred.^4^ Although approximately 80% of infected persons do not develop signs or symptoms, an explosive epidemic of microcephaly and Guillain-Barré syndrome has raised concerns about an etiologic association.^5^ On February 1, 2016, the World Health Organization announced that the cluster of neurologic disorders and congenital anomalies reported in Brazil constitutes a public health emergency of international concern.^3^ To the best of our knowledge, there are no data on Zika virus infection in patients with cancer. Here we report the first case.

A 58-year-old white woman, currently on treatment with anastrozol for a locally recurrent, hormone receptor–positive breast cancer, spent the New Year’s holiday in a coastal area of Sao Paulo State. In early January, she reported important tiredness, myalgia, headache, and retro-orbital pain. Two to 3 days later, she developed sudden episodes of high fever with intense sweating, which lasted 4 days. The patient sought medical assistance twice and was submitted to an extensive number of complementary examinations, the findings of which, besides a hemoglobin level of 10.0 g/dL (previously 13.5 g/dL), were normal. Approximately 10 days after the onset of the symptoms, the patient was referred to her oncologist in a tertiary center hospital, who noticed a mild abdominal maculopapular rash and ordered some serologic tests. Dengue virus immunoassays were negative, but indirect immunofluorescence assays for Zika virus demonstrated both immunoglobulin M (IgM) and IgG high titers. The patient is now recovered and had no neurologic symptoms.

This case illustrates some difficulties in diagnosing Zika virus infection in patients with cancer. As already highlighted in previous studies, cross-reactions with related flaviviruses are common and, unfortunately, Zika-specific tests are expensive and not widely available.^1^

Clinicians should be aware of the importance of including arboviruses as differential diagnoses of fever in patients with cancer, especially in epidemic areas. In doing so, patients would be less likely to be submitted to unnecessary and costly examinations. In addition, some patients with cancer who already have reduced platelet counts, secondary to the primary disease, chemotherapy, or other specific treatments, are at great danger of bleeding if not promptly advised not to take aspirin and nonsteroidal anti-inflammatory drugs if the infection is suspected. Also, a Guillain-Barré syndrome in a patient with cancer should be interpreted with caution in areas of the ongoing Zika virus transmission because it could easily be misdiagnosed as a paraneoplastic manifestation of the underlying disease.

The Zika outbreak is evolving rapidly and the expected number of new cases in patients with cancer should increase. The course of the disease in immunosuppressed persons and in patients presenting with significant comorbidities is still uncertain. Therefore, close follow-up is required to identify late or chronic symptoms.

## Adriana Hepner

No relationship to disclose

## Maria del Pilar Estevez Diz

**Travel, Accommodations, Expenses:** Mundipharma, Merck
